# Stroke-Induced Retinal Dysfunction: Mechanisms, Diagnostics, and Future Directions

**DOI:** 10.14336/AD.2025.0451

**Published:** 2026-07-31

**Authors:** Ting Wang, Zirun Feng, Jin Yang, Cuiying Liu, Heng Zhao, Sheng Huang

**Affiliations:** ^1^Department of Ophthalmology, Tongren People's Hospital, Tongren, Guizhou, China.; ^2^Beijing Institute of Brain Disorders, Laboratory of Brain Disorders, Ministry of Science and Technology, Joint Innovation Center for Brain Disorders, Capital Medical University, Beijing, China.; ^3^Medical College of Jishou University, Hunan, China.; ^4^Department of Radiology, Tongren People's Hospital, Tongren, Guizhou, China.; ^5^School of Nursing, Capital Medical University, Beijing, China.; ^6^Department of Neurology, Neurosurgery, and Ophthalmology, Tongren People's Hospital, Tongren, Guizhou, China.

**Keywords:** Stroke, Visual system, Neuroimaging, Retinal damage, Visual rehabilitation

## Abstract

Stroke has a profound effect on the retina's function, resulting in ischemia, nerve fiber thinning, microvascular issues, and breakdown of the blood-retina barrier. This leads to visual problems, including field defects, lower acuity, and damage to photoreceptors. In this comprehensive review, we delve into the historical milestones, diagnostic breakthroughs, and therapeutic approaches, with a particular focus on the retina as both a marker of and a target for addressing stroke-related dysfunction. Firstly, the retina is a non-invasive method for assessing cerebrovascular health, as it shares the same embryological origins and structural similarities with the brain. In addition, new imaging technologies, such as optical coherence tomography (OCT) and OCT angiography (OCTA), have revolutionized the way we detect alterations in the retina and their relationship to brain diseases. These developments have shown strong connections between retinal changes and cerebral pathologies, opening up new frontiers in our understanding and management of stroke-related retinal issues. Furthermore, new treatments, such as anti-inflammatory and antioxidant therapy, along with collaboration between doctors in other fields, including ophthalmology, neurology, and rehabilitation, demonstrate the importance of providing each patient with the proper care. At last, translational research, which connects biological discoveries with therapeutic uses, shows promise for improving early diagnosis, rehabilitation procedures, and outcomes for people who have had a stroke. Together, these advances position the retina as a powerful platform for understanding stroke pathology and developing innovative diagnostic and therapeutic strategies.

## 1. Introduction

Stroke is one of the most common causes of mortality and disability around the world. It has a robust effect on patients' quality of life, as it can cause various problems, including visual dysfunctions related to the retina [1, 2]. A stroke can cause problems with blood vessels and neurons that are especially detrimental to the retina, which is an extension of the central nervous system (CNS). Stroke-induced retinal dysfunction includes ischemia, microvascular abnormalities, nerve fiber thinning, disruption of the blood-retina barrier (BRB), and inflammation. These changes can cause visual problems, such as field defects and lower acuity [3], which make it harder to perform everyday tasks and maintain independence [4-6]. Even though these problems are very common, retinal dysfunction is often not detected in clinical practice. This oversight, together with an aging population and lifestyle changes that make strokes more common, makes the socioeconomic burden of stroke even worse, especially in low- and middle-income nations with few healthcare resources [7].

The retina's common embryological origin and anatomical similarities with the CNS make it a "window to the brain," which makes it a unique diagnostic tool. Because the retina and the brain share neurovascular structures, retinal abnormalities can often show problems in the brain. This makes the retina a non-invasive and straightforward way to check the health of the brain's blood vessels [8]. New imaging methods, such as optical coherence tomography (OCT) and optical coherence tomography angiography (OCTA), have made the retina even more helpful for diagnosis by allowing doctors to inspect microvascular alterations, nerve fiber thinning, and ischemic damage in high resolution [9]. These developments not only allow for early detection of stroke-induced changes but also aid in predicting stroke recurrence and guiding therapeutic interventions.

In this review, we provide a comprehensive exploration of the relationship between stroke and retinal dysfunction. We begin with a historical overview of research linking stroke and retinal dysfunction, followed by a brief review of the anatomical and physiological connections between the retina and the brain. Next, we examine the pathophysiological mechanisms underlying stroke-induced retinal damage. Additionally, we discuss the clinical applications of retinal evaluations in early diagnosis, stroke prediction, and rehabilitation planning. Finally, we outline future research directions aimed at advancing therapeutic strategies.

By integrating retinal and cerebral pathophysiology, we highlight retinal dysfunction as both a consequence of and a predictive marker for stroke. Through the lens of ophthalmology and neurology, we aim to offer insights that have the potential to improve research, clinical practice, and patient outcomes in this critical area of cerebrovascular health.

## 2. Brief research history

The retina is closely connected to the CNS, and its blood vessels and nerves are similar to those in the brain. This makes it a unique way to study the causes and treatments of stroke-related diseases. Over the years, considerable work has been done in understanding these links, which has led to improved methods for diagnosing and treating them (Fig. 1).

**Figure 1.**
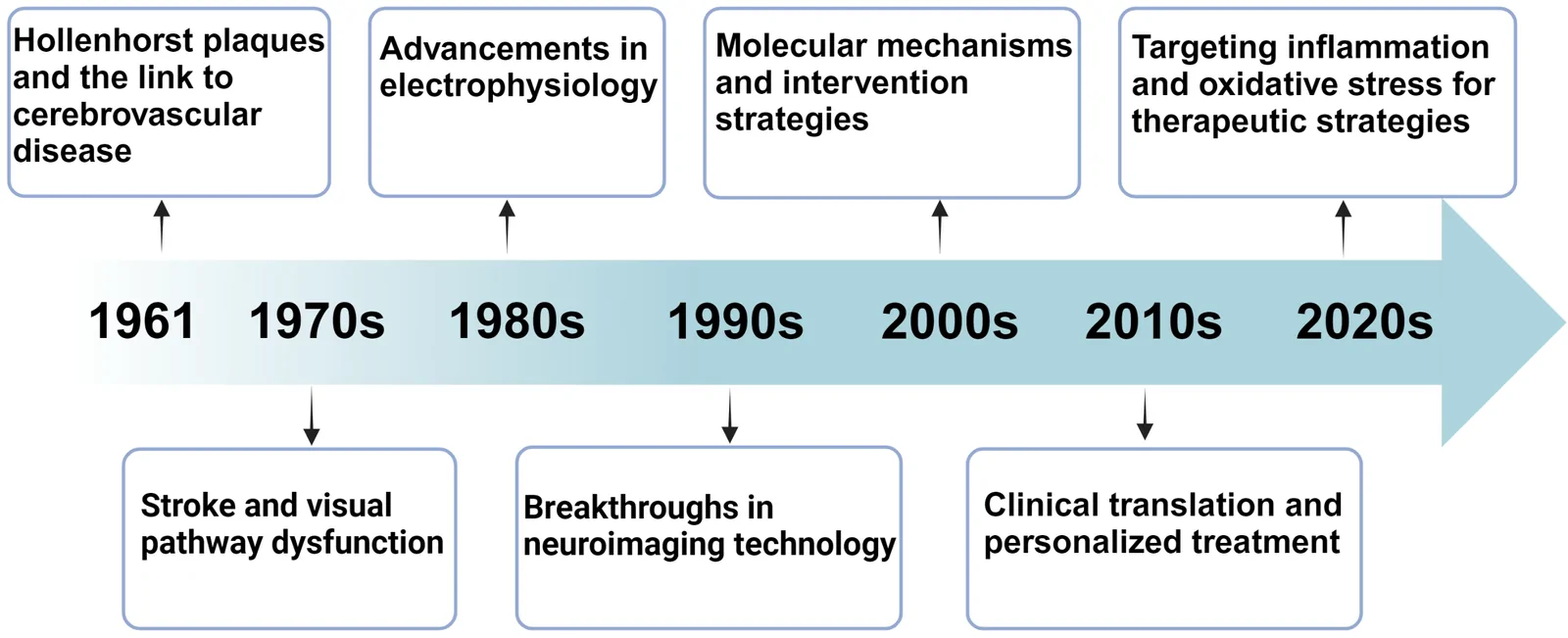
**Brief research history of stroke-induced retinal dysfunction**. This timeline highlights key advancements in understanding the relationship between stroke and retinal abnormalities.

Robert Hollenhorst's discovery of cholesterol emboli in the retinal arterioles in the 1960s marked a significant advancement [10]. These emboli, later known as "Hollenhorst plaques," demonstrated that the retina could be used to diagnose systemic vascular disorders. These plaques were known to be signs of atherosclerosis and were early signs of stroke risk. This led to the use of retinal exams in the diagnosis of cerebrovascular disease.

By the 1970s, the impact of stroke on the visual system gained more attention, with researchers documenting impairments such as spatial neglect [11], visual field deficits [12], and eye movement disorders [13]. These clinical observations emphasized the widespread influence of stroke on retinal and visual pathways. Electrophysiological tools, including visual evoked potentials (VEP) [14], have become instrumental in assessing disruptions in the visual pathways, further strengthening the connection between retinal and cerebral pathologies [15]. Additionally, the advent of fluorescein angiography during this period significantly improved the ability to detect retinal microvascular abnormalities, enabling earlier identification of ischemic changes [16, 17].

The 1980s witnessed the introduction of a wider range of methods, including improvements in electrophysiology. Research at this period showed that retinal ischemia caused ganglion cells to die through apoptosis and necrosis, which helped us understand how visual function loss happens [18]. VEP kept demonstrating how useful they were by showing that stroke patients had delayed signal transmission [19, 20].

In the 1990s, imaging technology made huge strides that helped us grasp the changing link between the retina and the blood flow in the brain. Magnetic resonance imaging (MRI) and computed tomography (CT) are two methods that have helped find ischemic lesions with great accuracy [21, 22]. At the same time, electroretinograms revealed how ischemia affected the inner retinal layers, providing scientists with insights into how neuronal loss causes vision problems [23, 24].

As we entered the 21st century, the focus changed to figuring out how stroke damages the retina at the molecular level. Studies showed that neurovascular coupling [25], inflammation [26], and oxidative stress [27] were all important factors in the course of ischemic retinal injury. Researchers found that high levels of inflammatory cytokines and reactive oxygen species were major causes of neuronal apoptosis and necrosis [28, 29]. Researchers are increasingly focusing on these molecular pathways, and anti-inflammatory and antioxidant medications have shown potential neuroprotective effects in laboratory models.

The past decade has witnessed remarkable innovations in technology and treatment strategies. OCT, which enables high-resolution imaging of retinal nerve fiber layers (RNFL) and retinal ganglion cells (RGCs), was a game-changer that complemented these other tools [30, 31]. OCTA has made it even easier to examine retinal microcirculation without the need for direct contact [32]. These improvements not only made diagnoses more accurate, but they also made the retina a more reliable indicator of brain vascular health. New ways to research molecular causes and test treatment options have come forth thanks to improvements in animal modelling, such as middle cerebral artery occlusion (MCAO) [33, 34] and oxygen-induced retinopathy (OIR) [35]. Also, the creation of specific treatments, including JAK/STAT signaling pathway inhibitors, has made it possible to treat retinal ischemia in innovative ways [36].

The use of artificial intelligence (AI) in retinal imaging has greatly improved the accuracy of diagnoses in recent years, allowing for the detection of subtle ischemia alterations with unmatched accuracy [37, 38]. The study shows that AI-enhanced OCTA image processing can be used to find ischemic strokes and classify their subtypes [39, 40]. New drugs, such as Baicalin [41] and pigment epithelium-derived factor [42], have shown a lot of promise for protecting the nervous system. This marks the beginning of a new era of precision therapy for retinal dysfunction caused by stroke.

From the initial discovery of Hollenhorst plaques to the development of cutting-edge molecular and imaging technologies, research in this field has transitioned from foundational observations to sophisticated clinical applications. The retina continues to give us important information about the health of the blood vessels in the brain, and it is a key part of improving diagnostics and treatments. In the future, researchers will probably focus on using interdisciplinary collaboration and new technologies to learn more about stroke-related retinal impairment and help patients recover.

## 3. Physiological Link Between Brain and Retina

To fully understand the complex interaction between the retina and the brain, we need to look at how they are both made, how they are similar in structure, and how they work together. The retina is a direct extension of the CNS and not only copies the brain's structure but also shows how it changes in response to disease. Table 1 shows how the retina and the brain are alike in many ways, such as how they develop from the same embryo, how their cells are organized, how they defend themselves, how they carry axons, and how they get blood. These similarities show how the retina can help us learn about brain health and disease, as seen in Figure 2. These links give us useful information about how retinal research can help us learn about brain health and disorders.

### 3.1 Embryological and Anatomical Similarities

The retina and the brain are deeply connected because they both come from the neuroectoderm during embryonic development. The neural tube turns into the CNS, which includes the brain and spinal cord, during development [43]. At the same time, an extension from this structure creates the optic vesicle, which eventually becomes the retina [44]. This shared developmental heritage not only connects the two structures but also explains the functional similarities between the retina and the brain.

Anatomically, the retina is like a little version of the CNS since it has layers of neurons and glia (Figure 2). The retina is composed of specialized neurons, including ganglion cells, and supportive glial cells, such as Müller cells. These cells are essential for keeping things in balance, controlling inflammation, and keeping the BRB intact [45]. These glial cells function and become ill in the same manner as their CNS counterparts, indicating that the retina is a direct extension of the brain's neuronal network [46].

The BRB and the blood-brain barrier (BBB) are comparable in both structure and function, which makes the retina an even better model for CNS research [47] (Fig. 2). The inner BRB is made up of endothelial cells that are closely connected by complicated junctions, a basement membrane, pericytes, and the pseudopodia of Müller cells. This is similar to the BBB. The retinal pigment epithelial (RPE) cells make up the outer BRB, which divides the choriocapillaris from the retina. These barriers keep the retina's milieu very controlled, which is important for neuronal function and immune privilege. The retinal barriers are comparable to the BBB in that they help keep the tissue intact [48].

**Figure 2.**
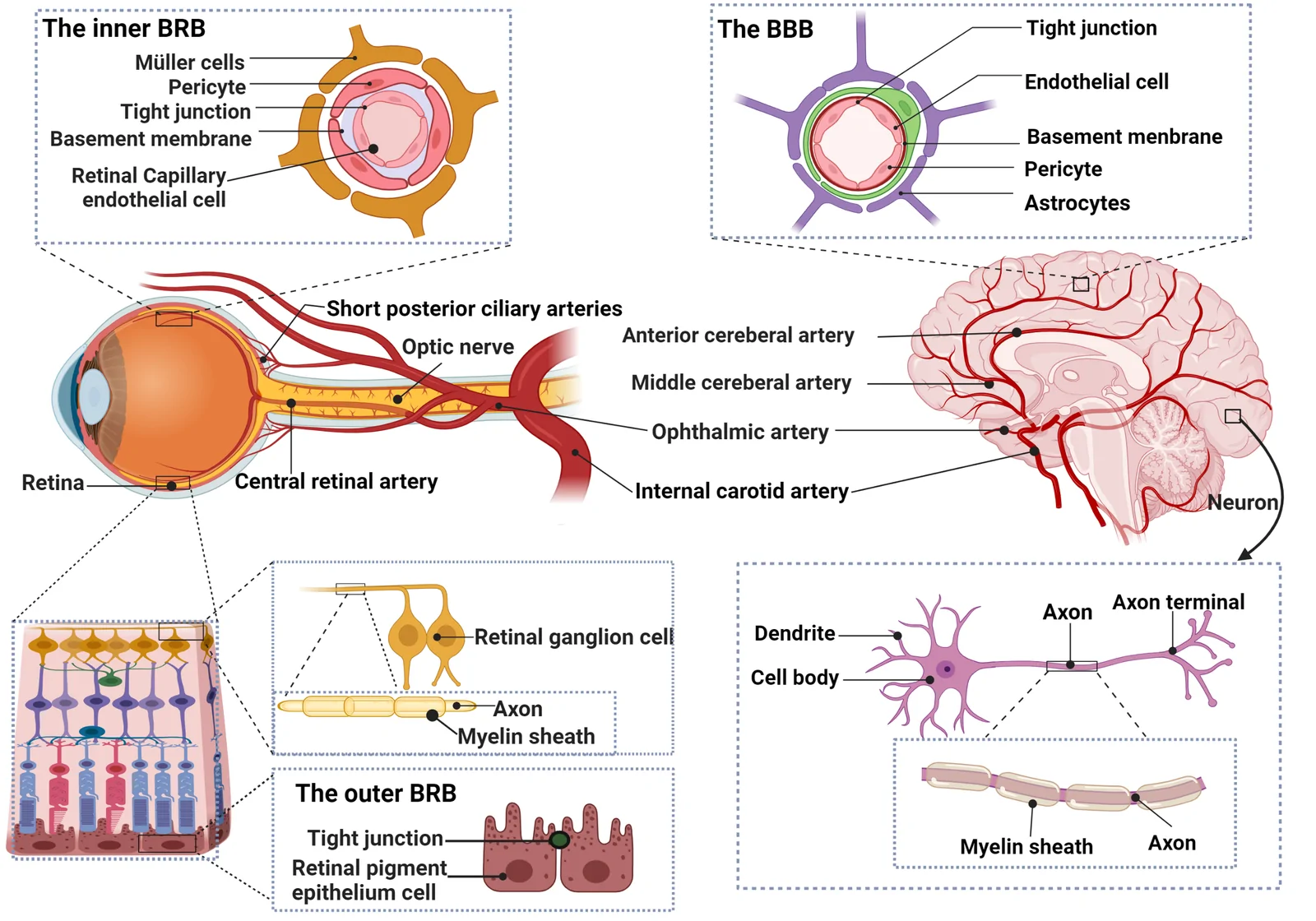
**Structural and Functional Connections Between the Retina and the Brain**. This figure illustrates the physiological link between the retina and the brain, highlighting their anatomical and vascular similarities.

The retina connects embryonic origins and anatomical structures, giving us a unique view of the brain. This allows us to comprehend, diagnose, and monitor CNS illnesses from a functional and clinically accessible point of view. Researchers and doctors can utilize retinal studies as a valuable tool to gain a deeper understanding of brain health and dysfunction, as they share common foundational knowledge.

### 3.2 Retinal Nerve Fiber Layer and CNS Axons

The RNFL, composed of axons originating from RGCs, shares many structural and functional characteristics with CNS axons (Figure 2). These axons bundle together to form the optic nerve, which directly transmits visual signals to the brain [49]. Beyond their structural similarities, both retinal and CNS axons rely on shared cellular and molecular mechanisms for their function and survival [50]. Both retinal and CNS axons are highly sensitive to neuroinflammation, oxidative stress, and vascular compromise [51].

### 3.3 Link Between Ocular and Cerebral Blood Supply

The ophthalmic artery and its branches are the main sources of blood for the eye (Figure 2). The optic nerve and the ophthalmic artery, which is the first branch of the internal carotid artery, both go into the orbit through the optic canal. The central retinal artery, which feeds the inner layers of the retina, and the short posterior ciliary arteries, which feed the choroid and the outer layers of the retina, are two of its most important branches [52].The ophthalmic artery also has other branches, such as the lacrimal artery and the supraorbital artery, which supply the lacrimal gland, eyelids, and other surrounding tissues [53, 54]. The choriocapillaris and the central retinal artery both provide a large blood supply to the retina, which has the highest metabolic demand of any tissue in the body [55].

**Table 1:** Comparison Between Retina and Brain.

Aspect	Retina	Brain
**Embryological Origin**	Develops from the neuroectoderm, an outgrowth of the neural tube, forming the optic vesicle.	Develops from the neuroectoderm, forming the CNS.
**Cellular Organization**	Layers of neurons and glial cells (e.g., Müller cells, astrocytes) maintain homeostasis and regulate inflammation.	Neurons and glial cells (e.g., astrocytes, microglia) perform similar functions in the CNS.
**Barrier Function**	The BRB regulates nutrient exchange and prevents leakage, resembling the BBB.	The BBB regulates the brain’s microenvironment, protecting it from toxins and pathogens.
**Axons and Nerve Fibers**	RNFL axons from retinal ganglion cells form the optic nerve, sharing mechanisms of axonal transport and sensitivity to damage with CNS axons.	CNS axons transmit signals across the brain, relying on similar mechanisms and are vulnerable to inflammation and oxidative stress.
**Blood Supply**	Supplied by the ophthalmic artery, with branches including the central retinal artery and choriocapillaris.	Supplied by the internal carotid and vertebral arteries, forming the Circle of Willis.
**Pathological Parallels**	Retinal changes (e.g., vascular damage, neuroinflammation) often reflect CNS pathologies.	CNS pathologies (e.g., neurodegeneration) are mirrored in retinal changes.
**Diagnostic Potential**	Non-invasive imaging (e.g., OCT, OCTA) reveals retinal microvascular and neuronal health, offering a window to brain health.	Neuroimaging (e.g., MRI, CT) assesses structural and functional brain health.

CNS: central nervous system; BRB: blood-retina barrier; BBB: blood-brain barrier; RNFL: retinal nerve fiber layers; OCT: optical coherencetomography; OCTA: optical coherence tomography angiography; MRI: Magnetic resonance imaging; CT: computed tomography

## 4. Evidence Supporting Stroke-Induced Retinal Dysfunction

This section consolidates key findings from epidemiological studies, imaging assessments, and experimental models to demonstrate the retina’s role as both a target and a biomarker in cerebrovascular disease.

### 4.1 The Clinical Epidemiology of Visual Impairments After Stroke

Homonymous hemianopia is the most prevalent sign of retinal dysfunction caused by a stroke, and visual field abnormalities are a common sign of this condition. The brain is where most of the damage happens, but the retina is also affected. This is because neuronal connections that are disrupted make it harder for the retinal layers that correspond to the afflicted vision field to interpret signals. The overall rate of visual impairment shortly after a stroke was thought to be 65%, with a range of 19% to 92%. Reports of visual field loss ranged from 5.5% to 57%, ocular motility issues from 22% to 54%, visual inattention from 14% to 82%, and impaired center vision from 70% to 70% [56]. Stroke-related alterations to the retina, like thinning of the RNFL and GCC, often make these problems worse [57]. In up to 50% of instances, partial recovery is achievable within six months. However, many patients still have problems that make it hard for them to get better and lower their quality of life [58].

Damage from a stroke can happen at different sites along the visual pathway, like the retina, optic nerve, and cortical areas. This can cause a range of problems, such as visual field defects, eye movement problems, and perceptual disorders [59] (Table-2). These problems with the visual system fall into three groups: afferent visual system dysfunction, higher cortical function deficits, and efferent visual system abnormalities [60].

Internal carotid artery stenosis can produce retinal hypoperfusion, which can lead to temporary vision loss or retinal claudication when exposed to strong light [12]. When blood clots from the carotid artery get into the ophthalmic artery, they can cause temporary monocular vision loss (TMVL) or amaurosis fugax. One serious effect of these emboli is central retinal artery occlusion (CRAO), which can cause sudden vision loss [61, 62]. Strokes that affect the optic chiasma, which is not common, cause bitemporal hemianopia because branches of the Circle of Willis supply it [60]. Lesions in the optic tract or occipital lobe often cause contralateral homonymous hemianopia. Visual field defects may not be symmetrical in the optic tract because the retinal nerve fibers are not entirely integrated. However, in the occipital cortex, the defects are usually symmetrical because the fibers are fully integrated [63].

**Table 2:** Stroke-Induced Visual Pathway Impairments.

Visual Pathway Location	Key Stroke-Induced Impairments	Clinical Observations and Implications
**Retina**	Retinal hypoperfusion causes transient visual loss or retinal claudication; emboli cause transient monocular visual loss (TMVL) or Central retinal artery occlusion (CRAO).	Significant impact on visual acuity; requires assessment of retinal blood flow and embolus management.
**Optic Nerve**	Vision loss due to emboli from carotid artery; progressive RNFL thinning due to trans-synaptic degeneration.	Progressive visual loss; importance of early detection and RNFL monitoring for long-term care.
**Optic Chiasma**	Bitemporal hemianopia due to Circle of Willis branch involvement.	Rare but diagnostically significant, requires imaging of Circle of Willis branches.
**Optic Tract**	Contralateral homonymous hemianopia; asymmetrical defects due to incomplete retinal fiber integration.	Visual field defects require tailored rehabilitation for asymmetrical impairments.
**Meyer’s Loop (Inferior Temporal Lobe)**	Contralateral superior quadrantanopia.	Specific to inferior temporal infarction; indicates focal brain damage with precise imaging utility.
**Superior Parietal Lobe**	Contralateral inferior quadrantanopia, often with hemineglect.	Requires cognitive and spatial rehabilitation strategies for improved quality of life.
**Central Occipital Lobe**	Contralateral hemianopia with macular sparing due to dual blood supply.	Typically preserves central vision, aiding differential diagnosis and rehabilitation planning.
**Brainstem**	Diplopia and nystagmus due to damage in eye movement control centers.	Indicators of brainstem stroke; emphasize early detection and motor rehabilitation.
**Right Hemisphere (Parietal/ Temporoparietal Junction)**	Spatial neglect, impacting perceptual and attentional networks, disrupting daily activities.	Highlights need targeted therapies to restore spatial awareness and perceptual capabilities.
**Unilateral Hemisphere**	Eyelid movement disorders, including difficulty in opening and closing, due to cortical motor control damage.	Rehabilitation focusses on motor control restoration; specific to cortical lesions in the right hemisphere.

TMVL: transient monocular visual loss; CRAO: central retinal artery occlusion; RNFL: retinal nerve fiber layers.

Patients who have had brainstem strokes often have eye movement problems such as diplopia and nystagmus. This highlights the importance of identifying these issues early [64, 65]. Spatial neglect is a perceptual and attentional disorder that occurs when patients fail to perceive objects on the left side of their visual field. It is commonly linked to strokes in the right hemisphere. Damage to the parietal and temporoparietal connections, for example, might cause this syndrome. It makes it hard to be aware of space and do everyday things like walking or eating [66, 67]. Routine cognitive tests often miss these problems, which shows that we need more specific diagnostic methods.

### 4.2 Retinal Microvascular and Neural Changes

Stroke has a robust effect on how the retina works, and there are direct changes in both the microvascular and neuronal components of the retina [68]. A lot of clinical and preclinical research has shown that retinal vascular parameters, such as caliber, tortuosity, and fractal dimension, not only show changes in the structure of the retinal microcirculation but also changes in the branching patterns and density of the retinal microvascular network (Table 3). The severity of this degeneration was connected to how long it had been since the stroke, which shows that the retina is a place where neurons die off over time because of brain damage caused by a stroke. These results support the idea that stroke-related damage to the brain's neurons might also affect the retina, where it can be seen as structural impairments.

Many investigations done on people and in hospitals have shown that stroke patients have typical problems with their retinal microvasculature [69]. A large case-control study involving 557 ischemic stroke patients found that their arteriolar and venular fractal dimensions were significantly smaller, their tortuosity was higher, their arteriolar caliber was narrower, and their venular caliber was wider than those of the controls. All of these changes were linked to a higher risk of stroke and stroke recurrence [70]. Other investigations found that retinal indications such as arteriovenous nicking, focal arteriolar constriction, hemorrhages, and exudates are linked to cerebrovascular events on their own [71, 72]. Retinal impairment is another effect of hemorrhagic stroke. Papilledema, which is swelling of the optic disc, can happen after a hemorrhagic stroke. This happens when the optic nerve's axoplasmic flow stops because of high intracranial pressure (ICP) [73]. OCT studies have indicated that the peripapillary RNFL thickness in these patients is higher, which is related to the level of ICP elevation [74-76]. If not treated, long-term papilledema can cause optic atrophy and permanent vision loss. Localized retinal ischemia may show up as flame-shaped hemorrhages or cotton-wool patches around the edges of the disc in some circumstances.

Stroke can cause many problems, but one of the most common is neural degeneration in the retina. This is especially true after an insult to the occipital lobe, which causes transsynaptic retrograde degeneration [77]. This process causes the loss of RGCs and the weakening of the macular ganglion cell complex (GCC), which gets worse over time. A study of 15 stroke patients with homonymous visual field abnormalities indicated that all of them had significant GCC thinning more than 2.5 years after their stroke. The thinning rates ranged from 1.99 to 5.68 µm per year [76].

### 4.3 Imaging Biomarkers and Animal Models of Stroke-Induced Retinal Dysfunction

Retinal imaging technology has shown that stroke causes structural and vascular damage in the retina that can be measured. OCT has shown that stroke survivors have a lot less RNFL and GCC, especially those who had occipital lobe infarctions and lost their visual field [76]. Studies using OCTA have shown that the density of blood vessels in both the superficial and deep retinal capillary plexuses is lower. They have also shown that the shape of the foveal avascular zone (FAZ) has changed, with less circularity and more eccentricity [78]. OCTA is a reliable method for detecting changes in the small blood vessels of the retina in stroke patients. These changes stay the same in macro-/microangiopathy strokes even after taking into account cardiovascular risk factors [79]. These alterations are linked to the type of stroke, its severity, and the duration since it began, which supports the use of retinal imaging as a diagnostic and predictive tool.

**Table 3:** Summary of Clinical and Experimental Evidence Supporting Stroke-Induced Retinal Dysfunction.

Evidence Type	Key Findings	Supporting Studies (Ref. No.)	Notes
**Retinal fundus photographs**	Reduced fractal dimension, arteriovenous nicking, focal arteriolar narrowing, hemorrhages, and exudates	Zhuo Y et al., 2017 [69]; Ong Y-T et al., 2013 [70]; De Silva DA et al., 2011 [71]; Yuanyuan Z, et.al 2020 [72];	Predict stroke risk and recurrence
**OCT Imaging**	RNFL and GCC thinning,	Yamashita T et al., 2020 [76]; Donaldson L et al., 2023 [77]	Reflects retrograde transsynaptic degeneration
**OCTA Imaging**	Reduced vessel density, FAZ shape changes	Shen Z et al., 2024 [78] ; Debourdeau E et al., 2025 [79]	Correlates with stroke subtype and severity
**MCAO Model**	Retinal perfusion reduction, inner retinal layer damage, reduced ERG a- and b-wave amplitudes	Allen RS et al., 2014 [80]; Justić H et al., 2022 [81]; Ritzel RM et al., 2016 [82]	Models acute ischemia and its effects on retinal function and structure
**UCCAO Model**	Chronic hypoperfusion leads to retinal thinning, gliosis, inflammation, and cell death	Lee D et al., 2018 [83]; Wagner N et al., 2021 [84]	Mimics chronic vascular insufficiency and progressive retinal degeneration
**Papilledema in Hemorrhagic Stroke**	Optic disc swelling due to elevated intracranial pressure (ICP)	Albanna W et al., 2021 [73]; Kalmar CL et al., 2022 [74]; Minakaran N et al., 2021 [75]	Detected via OCT as increased RNFL thickness
**ICH and SAH Models**	Retinal hypoxia, RGCs loss, and neovascularization via HIF-1α/VEGF-A pathway	Zhaba WD et al., 2021 [115]; Deji QZ et al.,2022 [116];	Demonstrates ischemic and inflammatory retinal injury in hemorrhagic stroke model

OCT: optical coherence tomography; RNFL: retinal nerve fiber layers; GCC: ganglion cell complex; OCTA: optical coherence tomography angiography; FAZ: foveal avascular zone; ERG: electroretinography; MCAO: middle cerebral artery occlusion; UCCAO: unilateral common carotid artery occlusion; ICP: intracranial pressure; ICH: intracerebral hemorrhage; SAH: subarachnoid hemorrhage; RGCs: retinal ganglion cells.

Animal models of cerebral ischemia provide us with clear insights into how strokes affect the retina. The MCAO model causes both cerebral and retinal ischemia, which leads to less blood flow to the retina, thinning of the inner retinal layers (including the ganglion cell and inner nuclear layers), ganglion cell death, activation of microglia, and lower a and b-wave amplitudes on electroretinography [80-82]. Reperfusion may help some people recover function, but long-term ischemia frequently causes permanent damage to the retina. The unilateral common carotid artery occlusion (UCCAO) paradigm shows the impact of chronic hypoperfusion, which includes gradual degeneration of the inner retina, gliosis, inflammation, and cell death [83, 84]. These models show that retinal injury is pathophysiologically connected to cerebral ischemia and give researchers a good way to study retinal pathology associated with stroke.

All of these clinical and experimental results show that the retina suffers from both primary ischemia damage because it shares blood vessels with the brain, and subsequent neurodegenerative alterations because of systemic inflammation and retrograde degeneration. This shows how important the retina is for both learning about how strokes work and finding non-invasive indicators and treatment targets.

## 5. The primary mechanisms responsible for post-stroke visual impairment

### 5.1 Neuroinflammation Response following Ischemic Stroke

When blood flow stops after an ischemic stroke, the afflicted brain tissues quickly become hypoxic, which starts a chain reaction of harmful events, such as the production of inflammatory cytokines, the activation of immune cells, and the breakdown of the BBB [85]. Microglia, which are the immune cells that live in the central nervous system, are some of the first to react to ischemia injury. They quickly become active in response to indications of damage in the area [86]. At the same time, astrocytes, which are very important for keeping the neurovascular unit healthy, are also activated to help with the immune response [87].

In addition to these indigenous glial cells, immune cells from outside the body, like monocytes, neutrophils, and T lymphocytes, are sent to the site of injury. These immune cells go into the ischemic brain tissue and release a number of proinflammatory cytokines and chemokines, including IL-1β, IL-6, and TNF-α. The release of inflammatory mediators makes local tissue damage worse by weakening the blood-brain barrier even more [88, 89]. The BBB breaking down lets immune cells get into the brain parenchyma, which starts a cycle of inflammation. Also, the influx of T lymphocytes makes the neuroinflammatory response stronger, which speeds up brain damage and death of neurons [90].

**Figure 3.**
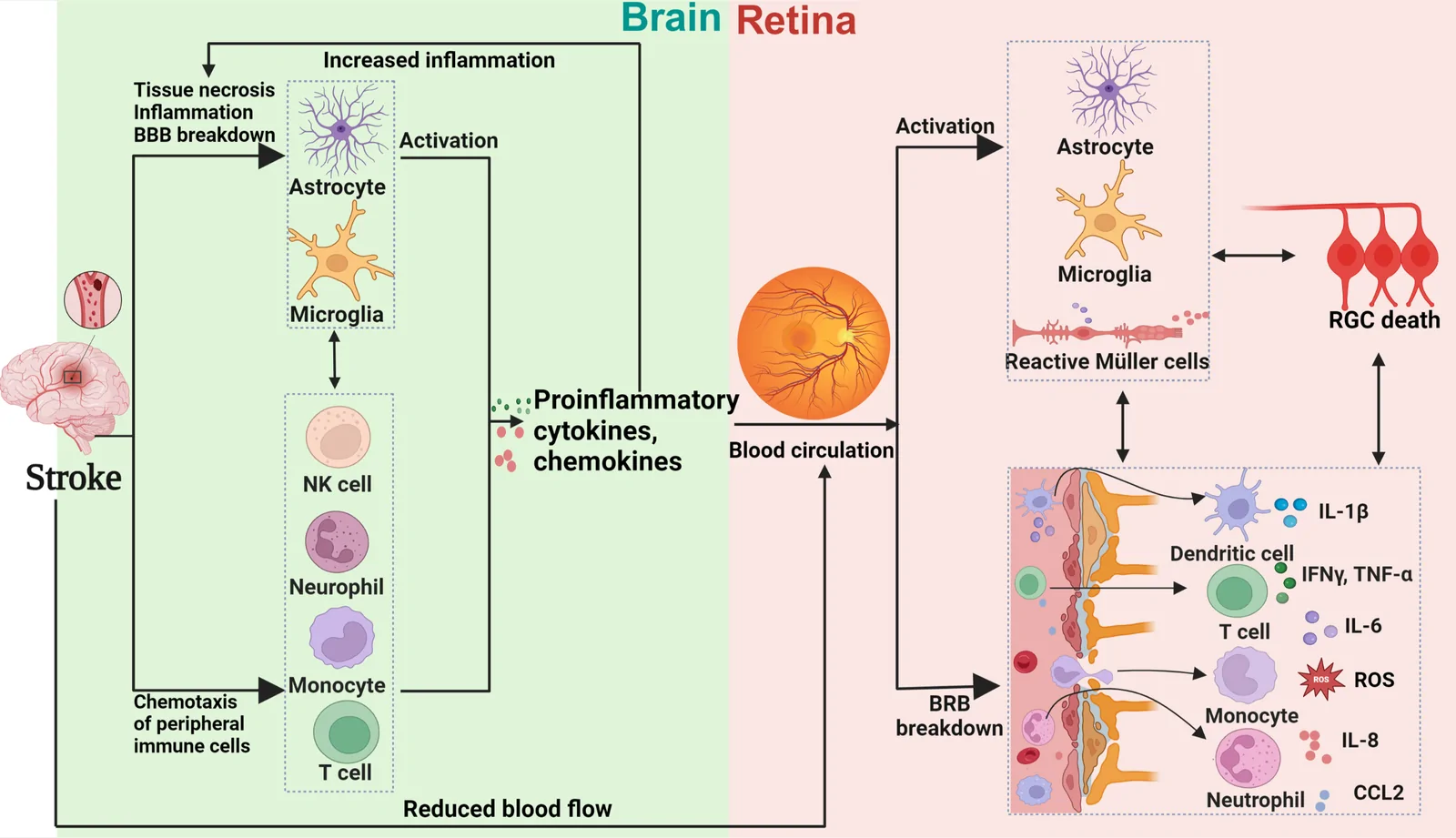
**Mechanistic model of stroke-induced retinal dysfunction mediated by brain-retina inflammatory crosstalk**. This schematic provides an integrated overview of how cerebral ischemia induces retinal injury through systemic and local inflammatory responses. Ischemic stroke promotes blood-brain barrier (BBB) disruption, glial activation, and the release of inflammatory mediators that circulate to the retina. There, they contribute to blood-retinal barrier (BRB) breakdown, microglial activation, and cytokine-driven oxidative stress, ultimately leading to retinal ganglion cell (RGC) loss and visual dysfunction.

## 5.2 Inflammation and Blood-Retinal Barrier Dysfunction in Stroke-Induced Retinal Ischemia

### 5.2.1 Disruption of Retinal Homeostasis by Inflammatory Responses and Oxidative Stress

Stroke or carotid artery stenosis, for example, can also cause less blood to flow to the retina, either directly or indirectly. This decrease is the first thing that sets off inflammatory and oxidative stress responses. Stroke causes systemic neuroinflammation that affects the retina indirectly, in addition to local ischemia damage [91]. Fibrinogen and other circulating DAMPs activate retinal glial cells, which quickly release proinflammatory cytokines (including IL-1β and TNF-α) and reactive oxygen species (ROS) [92-94]. These inflammatory mediators and ROS start normal pathways including NF-κB and NLRP3 inflammasome activation, which causes neuronal damage and pyroptosis [95, 96]. Infiltrating macrophages and neutrophils further intensify the inflammatory environment by producing additional cytokines and oxidative stress [97]. In addition to classical cytokine signaling, epigenetic modulators such as lncRNAs also play a key role in sustaining inflammation. Long non-coding RNAs (lncRNAs) such as H19 and 1810058I24Rik exacerbate inflammation by regulating mitochondrial dysfunction and inflammasome activation [98, 99]. Together, these local and systemic inflammatory cascades synergistically disrupt retinal immune homeostasis and structural integrity following stroke.

### 5.2.2 Stroke-Induced Breakdown of the Blood-Retinal Barrier

Owing to the structural and functional parallels between the BRB and the BBB, inflammatory mediators generated during stroke can also disrupt BRB integrity. Ischemic brain tissue releases various inflammatory mediators, including cytokines, ROS, and MMP-9. These factors enter the systemic circulation and contribute to BRB disruption [100, 101]. BBB breakdown facilitates infiltration of immune cells such as neutrophils and monocytes, which release NETs, cytokines, and ROS that further propagate inflammation [102, 103]. Single-cell RNA sequencing has identified elevated expressions of TNF, IFN-γ, and chemokines such as IL-6, CCL2, and CXCL10, which collectively impair BRB stability [104, 105]. Notably, BRB breakdown can occur within minutes after reperfusion. This process involves VEGFR-2-mediated degradation of tight junction proteins, including occludin and ZO-1, resulting in persistent vascular permeability [106]. Inhibition of VEGFR-2 signaling, for instance via bevacizumab, has been shown to preserve tight junction integrity and reduce vascular leakage [106, 107]. This oxidative milieu perpetuates canonical inflammatory cascades and exacerbates vascular injury.

### 5.2.3 Inflammation-Driven RGCs Apoptosis Post-Stroke

Prolonged disruption of the BRB leads to plasma protein extravasation, retinal edema, and hypoxia, all of which disturb vascular homeostasis and promote capillary occlusion. These vascular insults precede the degeneration of RGCs and thinning of the RNFL, with injury severity closely associated with ischemia duration [108, 109]. In parallel, systemic inflammation exacerbates retinal damage by inducing vascular edema, microvascular rarefaction, and progressive RNFL loss, as confirmed in both clinical and experimental studies [110-112]. These structural alterations impair inner retinal neuronal viability and contribute to irreversible visual dysfunction in stroke patients.

These interconnected mechanisms culminate in permanent loss of retinal structure and function, underscoring the need for early vascular and neuroinflammatory intervention in stroke-associated visual decline [113, 114].

### 5.3 Hemorrhagic Stroke and Potential Retinal Implications

Most stroke research on the retina has focused on ischemic causes, but new evidence suggests that hemorrhagic strokes, like intracerebral hemorrhage (ICH) and subarachnoid hemorrhage (SAH), may also have an effect on retinal health. These effects happen mostly because of high ICP, problems with the body's blood vessels, and neuroinflammatory pathways, not because of bleeding inside the eye [115].

Retinal hypoperfusion and inflammation caused by hemorrhagic strokes may make the retina less functional. Studies in the lab have demonstrated that SAH can cause inner retinal ischemia, which is marked by the loss of RGCs and an increase in hypoxia-inducible factor-1α (HIF-1α) and vascular endothelial growth factor (VEGF-A) [116]. These molecular alterations suggest that short-term drops in blood flow to the eye or spasms in the central retinal artery could make it harder for the retina to get oxygen, which could start neurodegenerative processes.

Another interesting process is trans-synaptic retrograde degeneration that happens after a hemorrhagic damage to the post-geniculate visual pathway, including bleeding in the occipital or lateral geniculate nucleus [117]. OCT imaging has shown that the RNFL and ganglion cell complex get thinner in patients with visual field deficits that match, which supports the idea that the retina can change its structure after a lesion to the central visual pathway, just like it does after an ischemic stroke [118].

In conclusion, there has not been as much research on how hemorrhagic stroke affects the retina as there has been on ischemic stroke. However, current clinical and experimental evidence suggests that high ICP, inflammation, ischemia, and systemic vascular injury can all be bad for the retina. Recognizing these mechanisms shows how important it is to include retinal examination in a whole stroke evaluation. More research is needed to figure out how far, when, and if retinal changes in hemorrhagic stroke can be reversed, as well as to look into their diagnostic and prognostic relevance.

## 6. Therapeutic Targets for Post-Stroke Retinal Protection

Researchers have found that inflammation and oxidative stress are major causes of damage to retinal neurons. This has led to the creation of specific treatments that aim to reduce retinal injury and protect neurons. Anti-inflammatory medicines, antioxidants, and new molecular targets are some of the most promising options. These treatments are at different stages of research, from preclinical studies to clinical applications. It is important to be able to tell the difference between these stages in order to judge their potential for translation.

### 6.1 Therapies with Clinical Evidence

Anti-inflammatory drugs that are already used in the clinic or for other purposes have shown promise in protecting the retina. Bevacizumab and other VEGF inhibitors are clinically approved and commonly used to treat disorders like diabetic macular edema and age-related macular degeneration. These drugs lower vascular permeability and swelling by keeping tight junctions intact, which is important for stroke-related retinal damage [118, 119]. In clinical ophthalmology, nonsteroidal anti-inflammatory drugs (NSAIDs) like indomethacin and corticosteroids have also been shown to work to reduce inflammation and vascular leakage. However, their use in stroke-related retinal pathology is still based on studies of other conditions [120].

Researchers have tried antioxidants like vitamin E and other free radical scavengers on people with different neurological and eye illnesses. They haven't been officially approved for protecting the retina after a stroke yet, but their capacity to reduce oxidative stress gives them a chance to be used in this way [121]. These treatments, especially when used with anti-inflammatory drugs, may help protect the structure and function of the retina in more than one way.

### 6.2 Therapies in Preclinical Development

Several new treatments are still in the preclinical stage. One example is the transient receptor potential melastatin 7 (TRPM7) channel, which has become a promising target for retinal damage caused by ischemia. After ischemia-reperfusion injury, TRPM7 levels rise in retinal Müller cells. This is linked to reactive gliosis and problems with vision [122]. In animal models, blocking TRPM7 with drugs greatly reduces damage to the retina, which suggests that it could be used to make drugs that help people [123].

Another experimental target is the NLRP3 inflammasome, which plays a big role in causing inflammation in retinal injury. This route makes neuroinflammation and cell death worse when it is turned on. Studies in mice have demonstrated that blocking NLRP3 can lower the activation of inflammasomes and protect retinal neurons after a stroke [124, 125]. These results support more research, although they are still far from being used in real life.

### 6.3 Regenerative and Precision Approaches

Stem cell-based therapies and other cell regeneration technologies are interesting ways to fix retinal tissue that has been damaged by ischemia. In preclinical models of optic nerve or retinal injury, delivering mesenchymal or dental pulp stem cells into the eye or under the retina has been demonstrated to preserve neurons [126]. These treatments are still being tested, though, and they have problems when it comes to using them in real life, such as how to get them to patients, how to keep the cells alive, and how to make sure they don't cause an immune response.

Gene therapy also has the potential to make neurons more resilient and lower inflammation, especially by changing important signalling pathways that are involved in oxidative stress and cell death [127]. One day, personalized medicine, which includes biomarkers and imaging data specific to each patient, may help sort people into groups for neuroprotective treatments that are right for them.

In short, there are a range of therapeutic techniques for protecting the retina after a stroke, from drugs that have been tested and authorized by doctors to new treatments that are still being tested in the lab. Clinical treatments like VEGF inhibitors and antioxidants can be used right away, while targets like TRPM7 and NLRP3 are intriguing but still in the early stages of development. Gene therapy and regenerative medicine provide us with a look into the future. It is important to clearly define the developmental stage of each therapy in order to set realistic goals and create a logical, step-by-step plan for improving the visual outcomes of stroke survivors.

## 7. Clinical Applications and Challenges

### 7.1 The Retina as a Window to the Brain: Insights into Stroke and Neurovascular Health

Because of how it looks and works, the retina has long been called a "window to the brain." The retina is a direct extension of the CNS and has the same embryological beginnings. It lets researchers see blood vessels and brain tissue without having to cut them open. This makes it a useful tool for researching systemic vascular and neural illnesses, including stroke [128].

Retinal imaging is a very significant way to check the health of the body's blood vessels. Researchers have found that alterations in the central retinal artery equivalent (CRAE) and venular equivalent (CRVE) are signs of cerebrovascular disease and can help predict stroke risk. There is a higher risk of both lacunar and cardioembolic stroke when there are problems with the retinal vessels, such as arteriovenous nicking, microaneurysms, and hemorrhages [129, 130]. These results show how the retina can indicate how healthy the blood vessels are and how likely someone is to have a stroke.

Retinal changes can also happen before clinical symptoms appear, which makes retinal imaging very beneficial for finding problems early in people at high risk, such as those with diabetes and high blood pressure. Reduced arteriolar-venular ratio (AVR), channel narrowing, and capillary occlusion are examples of retinal microvascular anomalies that can be early signs of cerebral small vessel disease, which is strongly linked to stroke recurrence [131-133].

New imaging methods like OCT, OCTA, and fundus fluorescein angiography (FFA) have made it easier to find small changes in the retina. These methods enable the visualization of subtle changes in blood vessels, such as when the density of the vessels decreases and the tortuosity of the vessels increases. OCTA, in particular, gives a full picture of the retinal microvasculature, which helps in making stroke prevention plans that are tailored to each person [78].

In conclusion, the retina's similarities in structure and function with the brain make it a powerful tool for diagnosing and treating strokes. Regular retinal exams can provide us with important information about the vascular health of our patients, allowing us to identify problems early and take steps to prevent them.

### 7.2 Therapeutics

Visual rehabilitation has become an important part of stroke care for patients with visual field deficits. Visual Scanning Training (VST) and Visual Field Rehabilitation Therapy (VFRT) are two important methodologies [134].

VST is a planned rehabilitation activity that helps people pay more attention to their vision and become more aware of their surroundings by making them intentionally scan the impaired side of their visual field. Patients gradually improve their ability to focus on the impaired visual field through repeated practice, which reduces the effects of their field loss [135]. VST is very helpful for individuals with homonymous hemianopia, as it enables them to find and see things more clearly in their daily lives. Studies show that VST greatly improves patients' capacity to do things like read and walk, which helps them get some of their perceptual abilities back in the damaged visual field, increases their confidence, and lowers their risk of falling [136]. However, the benefits of VST are different for each person. Some patients slowly lose the abilities they learned after stopping training, which shows how important it is to be consistent and persistent with VST.

VFRT, on the other hand, utilizes visual stimulation to activate dormant brain pathways. Its goal is to restore or improve visual functions near the edge of the visual field. VFRT usually includes a series of visual stimuli and tracking exercises that activate healthy neurons near the damaged visual field. This helps the brain become more adaptable and flexible [137]. The purpose of this therapy is to help the patient see better in their peripheral vision, which will widen their overall field of vision. Research has indicated that VFRT can improve some people's ability to see, although its effectiveness varies greatly, and in certain situations, recovery is restricted. VFRT frequently involves a lot of training, which means that patients have to stick with it. Some patients may lose motivation because visual rehabilitation activities can be boring and not always effective. To keep patients positive and persistent in clinical settings, phased goals and psychological support are needed [138].

Visual scanning training and visual compensation therapy are very important for stroke patients with visual field deficits. For some, they make a big difference in how well they can see and how well they can do everyday tasks. However, there are still issues with adherence, creating tailored plans, and insufficient resources. Future goals include incorporating neuroimaging data to enhance tailored rehabilitation programs, increasing the likelihood of patients adhering to their plans, and augmenting psychological support to make visual rehabilitation more effective in clinical settings.

## 8. Future Research Direction

To better understand stroke-related vision loss and improve clinical outcomes, future research should focus on a few critical areas. Long-term, large-scale clinical trials are needed to find out how different types of strokes, such as ischemic and hemorrhagic, affect the visual system in different ways. This research will assist in finding groups of patients who are at high risk, keep track of how their vision problems get worse, and improve individualized rehabilitation plans. It is essential to monitor the progress of vision recovery to assess the effectiveness of existing treatments and optimize clinical interventions for maximum success.

Because stroke-related vision problems are so complicated, they need to be looked at from many different fields. Ophthalmologists can check for and treat damage to the retina and optic nerve, whereas neurologists look at the bigger picture of how a stroke affects the whole body. Rehabilitation professionals play a crucial role in creating tailored visual rehabilitation exercises, providing psychological support, and implementing comprehensive intervention programs. This collaborative approach will help create personalized rehabilitation strategies that will lead to the best possible recovery outcomes.

Translational research is important for connecting what scientists find in the lab with what doctors can use in the real world. For example, medications that focus on the integrity of retinal microvessels or change neuroinflammation could be tested to see if they can restore vision. Researchers and doctors will need to collaborate to translate these discoveries into effective treatments that benefit patients immediately.

Additionally, combining artificial intelligence (AI) and digital tools has significant potential to enhance stroke care. AI systems can look at retinal and cerebral imaging data to find small changes, guess when a stroke may happen again, and keep an eye on visual recovery. This makes diagnoses more accurate and allows for more individualized rehabilitation regimens. AI-driven methods will change how care is delivered by using patient-specific data, which will lead to better therapeutic outcomes.

Discipline can make a lot of progress in treating vision problems caused by strokes by combining clinical research, new technologies, collaboration between different fields, and translational studies. These combined efforts are meant to safeguard and restore vision, which will make life better for stroke survivors. Personalized medicine and AI-enhanced diagnostics are intriguing new ways to care for stroke patients. They make real-world improvements for patients as research makes progress.

## 9. Summary and Conclusion

Stroke-induced retinal dysfunction shows how closely the retina and brain are connected. They have comparable embryological beginnings and structures, which makes retinal studies a good way to learn more about cerebrovascular health. We looked into how research has changed over time, how diagnostic technology has improved, and how stroke can damage the retina. The most important discoveries showed that neurovascular disturbances, inflammatory reactions, and BRB breakdown all play a role in visual impairments. At the same time, new imaging methods like OCT and OCTA have changed the way we undertake non-invasive diagnostics.

New treatments that focus on inflammation, oxidative stress, and neurodegeneration are making it possible to create tailored treatments. Collaboration between ophthalmology, neurology, and rehabilitation is essential for creating complete treatment plans, and translational research is linking what we learn in the lab with what we do in the clinic. Combining AI with new treatment methods could help stroke patients find their vision problems earlier, get better faster, and stay better for longer.

Future research should focus on large-scale clinical studies, molecular insights, and technological innovations to deepen our understanding and improve patient outcomes. By advancing the integration of diagnostics and therapeutics, we can provide stroke survivors with better protection and restoration of visual function, significantly enhancing their quality of life.
